# Special Issue: Asymmetric Synthesis 2017

**DOI:** 10.3390/molecules22091504

**Published:** 2017-09-08

**Authors:** Rafael Chinchilla

**Affiliations:** Departamento de Química Orgánica, Facultad de Ciencias, and Instituto de Síntesis Orgánica (ISO), Universidad de Alicante, Aptdo. 99, 03080 Alicante, Spain; chinchilla@ua.es

The use of asymmetric synthetic methodologies plays a crucial role, nowadays, in the preparation of bioactive or other interesting compounds. Developments in this fast-moving area are constant, from the classical direct modification of naturally found chiral molecules to the use of catalyzed reactions using metal complexes bearing chiral ligands or chiral metal-free organocatalysts. This Special Issue aims to collect research and review articles showing significant and remarkable examples of the use of all these different methodologies to access the asymmetric synthesis of compounds of interest.

The review by Jo et al. shows, with recent examples, how different natural products can be prepared in a stereoselective fashion by means of asymmetric cyclization reactions, where the stereoselectivity of the reaction is controlled by the asymmetry in the substrate [1]. The starting chiral substrates are frequently obtained from the chiral pool. Fekete et al. show in their research article how enantiomeric bicyclic pyrrolo-pyrimidines can be obtained by transferring the chirality from norbornene derivatives using a domino ring-closure protocol [2]. In this case, both enantiomers of the initial norbornene derivatives were obtained by the old but reliable resolution of a racemate. Kaplan et al. show another example of transferring the chirality of an enantiomerically enriched starting material, in this case a chiral Hajos-Parrish ketone, for a stereocontrolled approach to the *cis*-decalin framework of clerodane diterpenes and biologically active quinone sesquiterpenes, a process also featuring a highly regioselective diazoalkane-carbonyl homologation reaction [3].

The generation of stereoselectivity using chiral catalysts is the most frequently used procedure in asymmetric reactions. In a catalytic asymmetric reaction, a chiral catalyst is used to produce large quantities of an optically active compound from a precursor that may be chiral or achiral. Synthetic chemists have developed numerous catalytic asymmetric syntheses that convert prochiral substrates into chiral products with high enantioselectivity.

Many of these methodologies are based on the use of metal complexes bearing chiral ligands as catalysts. Therefore, the development of new chiral complexes or new uses for old ones is challenging, and this Special Issue includes several interesting examples. Thus, the article by Liu et al. shows a practical asymmetric synthesis of the bioactive fatty acid metabolite (*R*)-matsutakeol and analogs. The process is based on the asymmetric addition of terminal alkynes to aldehydes catalyzed by chiral zinc complexes, achieving excellent enantioselectivities [4]. Fujiwara et al. have developed an expedient and efficient asymmetric total synthesis of both natural antifungal (*R*)-podoblastin-S and (*R*)-lachnelluloic acid, the crucial step of the methodology being an enantioselective Mukaiyama aldol reaction catalyzed by a chiral titanium-based complex [5]. Kraft et al. show in their research article the preparation of two novel carbohydrate-derived oxazolines and their application as chiral ligands in a highly enantioselective palladium-catalyzed Tsuji-Trost allylic alkylation reaction [6].

In recent years, the use of purely organic metal-free chiral catalysts in asymmetric synthesis has been gaining significant attention due to some advantages compared to the use of metal complexes as catalysts, such as their typically higher stability or lower ecological footprint. These asymmetric organocatalyzed reactions are also represented in this Special Issue. Thus, the review of Alonso et al. covers recent developments in the organocatalyzed asymmetric conjugate addition of carbon and heteroatom nucleophiles to nitroalkenes, a very interesting synthetic tool for the construction of highly functionalized synthetic building blocks [7]. The research article by Wang et al. shows an enantioselective Michael addition between cyclic β-diones and α,β-unsaturated enones organocatalyzed by a quinine-based primary amine or squaramide [8].

This Special Issue illustrates how the research on the use of all the different methodologies employed for achieving asymmetric synthesis is nowadays quite active. However, much work must be done. Despite all the developments obtained until now, more efficient and stereoselective methodologies, applicable to more substrates, need to be developed. There is no doubt that asymmetric synthesis will continue to be a crucial research area in the coming years.
